# miRNA101a secreted by EATMs regulates atrial fibrillation through the PDGF-mediated PI3K/AKT pathway

**DOI:** 10.3389/fphar.2026.1725208

**Published:** 2026-04-16

**Authors:** Zheng Sihao, Li Xiaoliang, Yue Honghua, Qin Xiaoli, Jiang Daisong, Zhang Zheng, Liang Weitao, Wu Zhong

**Affiliations:** 1 Department of Cardiovascular Surgery, West China Hospital, Sichuan University, Chengdu, Sichuan, China; 2 Department of Cardiothoracic Surgery, The first People’s Hospital of Neijiang, Neijiang, China

**Keywords:** atrial fibrillation, exosome, macrophage, miRNA101a, myocardial fibrosis

## Abstract

**Background:**

Atrial fibrillation (AF) is the most prevalent cardiac arrhythmia worldwide, and microRNAs (miRNAs) are gaining significant attention in cardiovascular disease research. In this study, we explored the specific mechanisms by which miR101a contributes to the pathogenesis of AF, aiming to identify a novel biomarker for its early detection.

**Methods:**

In this study, we examined the differential expression of miR101a in human left atrial appendage tissue using RT-PCR. We developed a Sprague-Dawley rat model of AF and assessed the expression of associated phenotypic markers and proteins through flow cytometry, immunofluorescence, and Western blotting. Potential targets were identified via bioinformatics analysis and dual luciferase assay. We modulated miR101a expression using adenoviral transfection to elucidate its regulatory mechanism in AF. This approach allowed us to identify and validate the pathway through which miR101a influences AF.

**Results:**

The experimental results indicated that miR101a was highly expressed in the sinus rhythm group of patients and played a crucial role in the myofibrosis associated with AF. miR101a interacted with PDGF-DD, contributing to fibroblast fibrosis, and modulated the fibrotic process by promoting the degradation of collagen and extracellular matrix in AF. *In vivo* animal experiments demonstrated a protective role of miR101a in the progression of AF. Furthermore, the findings revealed that the PI3K-Akt pathway was activated in AF, and miR101a was capable of modulating AF progression through this pathway.

**Conclusion:**

In this study, we demonstrated that miR101a, secreted by epicardial adipose macrophage-derived exosomes, regulates AF via the PI3K-Akt pathway and by targeting PDGF-DD. These findings suggest that miR101a holds promise as a novel biomarker for AF.

## Graphical Abstract

**Figure:**
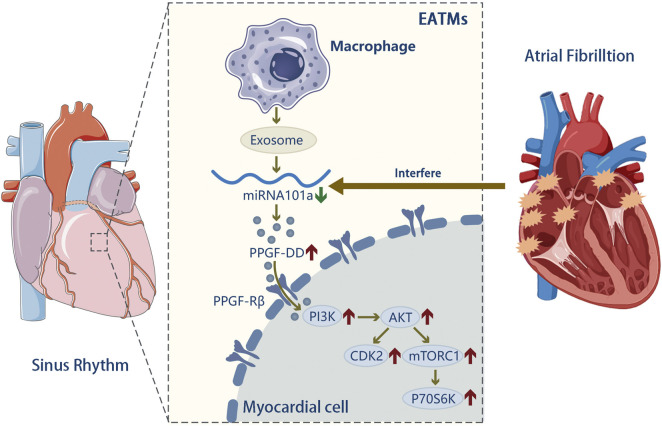

## 1 Introduction

Atrial fibrillation (AF) represents approximately one-third of all cardiac arrhythmias and impacts the health of tens of millions globally, with a lifetime prevalence reaching up to 25% among middle-aged adults (Brundel et al., 2022). The rising costs associated with AF treatment and management exert substantial social and economic pressures (Joglar et al., 2024). In the face of this increasingly severe public health challenge, gaining a deep understanding of the pathogenesis of AF has become crucial for effective prevention and treatment. Current research categorizes AF as a form of atrial cardiomyopathy (ACM), wherein cardiomyopathy manifests in the atria, offering a crucial pathogenetic substrate for AF’s onset and progression (Sagris et al., 2021; Andersen et al., 2021). Although the precise pathogenesis of AF remains unclear, it is closely associated with atrial remodeling (Lau et al., 2019). Both electrical and structural remodeling of the atria contribute to this process. Myocardial fibrosis disrupts the balance between degradation and synthesis of the extracellular matrix (ECM), leading to the excessive proliferation of cardiac fibroblasts and exacerbating cardiac structural remodeling. The fibrous tissue’s electrically insulating properties hinder normal signal propagation in the atria, causing discontinuity and heterogeneity in electrical conduction. This abnormal conduction results in multiple micro reentrant loops, ultimately triggering atrial fibrillation (Schotten et al., 2011; Kong et al., 2014). These pathophysiological effects contribute to the foldback mechanism by interfering with electrical signal conduction.

Given that myocardial fibrosis plays a central role in triggering and sustaining AF, identifying the key molecules regulating this pathological process is of paramount importance. Recent studies highlight miR101a’s significant regulatory role in pathological fibrosis, positioning it as a target for fibrosis treatment (Park and Kang, 2020; Wang et al., 2020; Li et al., 2021a; Huang et al., 2017). Platelet-derived growth factor (PDGF) is among the growth factors that regulate cellular processes such as proliferation, differentiation, apoptosis, and migration (Zhao et al., 2013; Heldin and Westermark, 1989; Leask, 2010; Heldin and Westermark, 1999). PDGF-DD (Platelet-Derived Growth Factor DD), in particular, plays a pivotal role in post-infarction myocardial fibrosis progression, acting through autocrine and paracrine pathways (Zhao et al., 2013). Our research confirmed that miR101a targets PDGF-DD in SD rat cardiac fibroblasts, suggesting PDGF-DD’s key role in the phenotypic transition of cardiac fibroblasts to myofibroblasts, thereby promoting migration, proliferation, and collagen synthesis, aggravating myocardial fibrosis.

However, the development of myocardial fibrosis is not an isolated event involving fibroblasts; inflammatory cells within its microenvironment, particularly macrophages, also participate in the process. Macrophages, differentiated from blood monocytes, are vital to vertebrate immunity, both specific and non-specific (Gentek et al., 2014). Epicardial adipose tissue macrophages (EATMs) are crucial immunomodulatory cells within epicardial adipose tissue, releasing inflammatory cytokines such as TNF-α, IL-6, IL-1β, and MCP-1, which are implicated in AF’s inflammatory pathogenesis (Fan et al., 2023; Sacks and Fain, 2007). It is noteworthy that a complex communication mechanism exists between these immune cells and fibroblasts, potentially influencing fibroblast function by secreting vesicles carrying regulatory molecules.

In summary, we hypothesize that miR-101a contributes to AF-induced myocardial fibrosis by targeting PDGF-DD to drive phenotypic changes in cardiac fibroblasts, while also potentially being delivered by epicardial adipose tissue-derived macrophages via paracrine exosomes to suppress PDGF-DD expression and regulate fibroblast activity.

## 2 Materials and methods

### 2.1 Human samples collection

Human left atrial appendage (LAA) samples were collected from patients with and without AF at the cardiovascular surgery department of West China Hospital, Sichuan University, China (each group n = 15), similarly, we collected preoperative peripheral venous blood from AF patients and SR patients at the cardiovascular surgery department of West China Hospital, Sichuan University (AF Group n = 16, SR group n = 18). The diagnosis of AF was established based on documentation by either a surface electrocardiogram or a single-lead electrocardiogram recording device, with episodes lasting more than 30 s. Atrial tissue samples were obtained from participants who provided informed consent, either personally or through their family members. All experimental procedures were approved by the Ethics Committee of West China Hospital, Sichuan University, and were conducted in strict accordance with the principles outlined in the Declaration of Helsinki.

### 2.2 Experiments reagents

Antibodies for CD86 (374,202) and CD206 (321,102) were obtained from BioLegend; primary antibodies PDGF-D (40–2100; PA5-118148; Invitrogen), total-PDGFRβ (3169; CST), phosphorylated-PDGFRβ (3161; CST), Col1a1 (91144S; CST), Col3a1 (30565S; CST), total-PI3K (ab302958, Abcam), phosphorylated-PI3K (4228T; CST), total-Akt (9272; CST), phosphorylated-Akt (9271T; CST), totalCDK2 (2546T; CST), phosphorylated-CDK2 (2561S; CST), total-P70S6K (2708T; CST), phosphorylated-P70S6K (9234T; CST), FSP-1 (ABclonal; A19109), Vimentin (10366-1-AP; Proteintech), a-SMA (14395-1-AP; Proteintech), α-tublin (14555-1-AP; Proteintech) and GAPDH (60004-1-Ig; Proteintech), CD63 (ab134045; Abcam), CD81 (ab79559; Abcam), TSG101 (ab125011; Abcam), Calnexin (ab22595; Abcam). The blots were probed with HRP-conjugated secondary antibodies (ZB-2301/2305; ZSGB-BIO), PI3K inhibitor LY294002 (HY-10108), PDGF-DD (HY-P702837) and Angiotensin II (HY-13948) were purchased from MCE; miRcute miRNA Isolation Kit was peichused from Tiangen biochemical technology Co., Ltd (DP501). The miR101a-shRNA adenovirus was packaged by Shanghai Hanheng Biotechnology Co., Ltd,PureBinding® RNA Immunoprecipitation Kit purchased from China Geneseed Bio (P0101).

### 2.3 Animals

Male Sprague-Dawley (SD) rats, aged 6–8 weeks and weighing 250 ± 10 g, were obtained from Chengdu Dashuo Laboratory Animal Co., Ltd. They were housed in a specific pathogen-free facility, with unrestricted access to food and water, and were handled in accordance with the Institutional Animal Care and Use Committee guidelines. This study was approved by the Animal Ethics Committee of West China Hospital, Sichuan University. All animal experiments were conducted at the Animal Experiment Center of the Laboratory Animal Center, West China Campus, Sichuan University.

### 2.4 Western blot

Tissues or cells were rinsed with cold phosphate-buffered saline (PBS) and lysed in RIPA buffer supplemented with a protease inhibitor cocktail. Following ultrasonication, cell debris was removed by centrifugation at 12,000 g for 15 min at 4 °C. The protein concentration in the resulting lysates was measured using a bicinchoninic acid assay. Equal amounts of protein were separated by 8%–12% SDS-PAGE (Sodium Dodecyl Sulfate Polyacrylamide Gel Electrophoresis) and transferred to polyvinylidene difluoride (PVDF) membranes (Merck Millipore). The membranes were then blocked with 5% skimmed milk in PBST buffer and incubated overnight at 4 °C with primary antibodies. After washing, membranes were treated with IRDye-800CW-conjugated goat anti-mouse IgG or goat anti-rabbit IgG secondary antibodies. Visualization was performed using a ChemiDoc XRS system (Bio-RAD), and the grayscale values of each band were quantified using accompanying software. All procedures were repeated in at least three independent experiments.

### 2.5 Histological analysis

Left atrial (LA) tissue sections from patients or rats were embedded in paraffin and sliced into 5-µm-thick sections. Hematoxylin and eosin (HE) staining was used to assess the degree of inflammatory infiltration in the LA, while Masson’s trichrome staining was employed to evaluate fibrosis levels. Sections were examined under a microscope, and data were quantified using ImageJ software, all images were quantified using the ImageJ software. Five visual fields were randomly selected for each sample, and the fluorescence intensity or an average number of positive cells were calculated. Immunofluorescence analysis was conducted to identify macrophages and fibroblasts, using antibodies against FSP-1(Fibroblast-Specific Protein 1), vimentin, α-SMA, CD86, and CD206(Specific antibody information is detailed in Section 2.2).

### 2.6 Transwell

Cells in optimal growth conditions were subjected to starvation treatment by adding serum-free medium for 24 h. A mixture of matrix gel and medium was then introduced into Transwell chambers and incubated at 37 °C for 1–3 h. Following this, 100 µL of serum-free culture medium was added, and the plates were further incubated at 37 °C for 30 min to allow hydration. After digestion and starvation, cells were resuspended in serum-free medium, adjusting the cell density according to the number of cells to be inoculated in the upper chamber. The 24-well plates were incubated at 37 °C with 5% CO2 and 90% humidity for 24–48 h. To fix the cells, 600 µL of 4% paraformaldehyde was added to each well for 30 min. The chambers were then placed in 24-well plates and stained with 600 µL of crystal violet staining solution for 10 min, followed by washing with PBS. After air drying, qualitative analysis was conducted under a microscope, with images captured from three to five fields of view. These images were averaged for quantitative analysis using ImageJ software.

### 2.7 Animal models of atrial fibrillation

SD rats were subcutaneously administered Angiotensin II (Ang-II) to establish an animal model simulating AF. Forty rats were randomly assigned to four experimental groups (n = 10 per group) as follows:Blank group: Rats were maintained under standard conditions without any intervention, with free access to food and water; Ang-II group: Rats received subcutaneous injections of Ang-II to induce AF; Ang-II + miR101a-shRNA group: Rats received concurrent subcutaneous injections of Ang-II and an adenovirus carrying sh-miR101a; miR101a-shRNA group: Rats underwent *in situ* injection of homologous random sequence shRNA adenovirus into the LAA:Upon opening the pericardium, *in situ* injections were performed into the LAA of rats under microscopic guidance. Three random sites on the surface of the LAA were selected, and 15 μL of the viral solution was injected per site. Care was taken to deliver the viral vector into the parenchyma of the LAA, avoiding superficial or intracavitary administration, which could lead to unsuccessful transfection. Throughout the procedure, vital signs were closely monitored, and any signs of bleeding were promptly assessed. After each injection, gentle pressure was applied to the puncture site using a sterile cotton swab.Ang-II was dissolved in sterile physiological saline and stored at 4 °C. Using a 2.5 mL syringe, Ang-II was administered subcutaneously at a dose of 7.2 mg/kg per rat. Injection sites were alternated among the interscapular region, abdomen, and inner thigh daily for 14 consecutive days. The general condition of the rats was monitored regularly, and electrocardiogram (ECG) recordings were performed twice daily. If signs of redness, swelling, or induration were observed at the injection site, the location was changed and disinfected with medical iodine solution. After 14 days of subcutaneous injections, AF was induced via transesophageal electrode stimulation in all groups.

After 14 days, Sprague Dawley (SD) rats were anesthetized and subjected to tracheal intubation for assisted ventilation. Continuous monitoring of heart rate was conducted using a BL-420 electrophysiological recorder to capture surface electrocardiograms. A 10F catheter equipped with stimulation electrodes was introduced orally into the esophagus. The catheter’s position was finely adjusted based on the esophageal electrogram recorded by the electrodes, ensuring that the electrogram exhibited a prominent upright atrial A wave (R-type or Rs-type) and a superficial inverted ventricular V wave (Qr-type or QS-type). To assess cardiac function, S1S1 and S1S2 pacing protocols were employed to determine pacing thresholds, sinoatrial node recovery time (SNRT), sinoatrial conduction time (SACT), atrioventricular nodal refractory period (AVNRP), and atrial effective refractory period (AERP). Subsequently, incremental pulse stimulation (ranging from 25 to 83 Hz, with a duration of 30 s, repeated twice at each frequency with 5-min intervals) was administered to the rats to induce AF. An episode of AF was characterized as a rapid arrhythmia lasting longer than one second, marked by variable A wave amplitude and spacing, along with irregular RR intervals.

### 2.8 Macrophage extraction from epicardial adipose tissue

Under aseptic conditions, LAA tissue was collected and rinsed 2–3 times with pre-cooled PBS. Adipose tissue was meticulously separated using ophthalmic scissors and cut into small fragments. These fragments were then immersed in 2 mL of isolation buffer and transferred to a 15 mL centrifuge tube. The tube was incubated in a shaker at 37 °C for 45 min to facilitate digestion. Following digestion, the tissue solution was filtered through a 70 µm filter, and the resulting cell suspension was centrifuged at 4 °C at 300 g for 8 min. The floating tissue and supernatant were carefully aspirated using a syringe. The cell pellet was resuspended in pre-cooled flow-through buffer, and the cell density was adjusted to1 × 10^6^/mL for future use.

### 2.9 Ultracentrifugation for EATM-Exo extraction

The isolated macrophages from step 2.8 were seeded in complete culture medium and incubated at 37 °C in a 5% CO_2_ incubator. Upon reaching 70%–80% confluence, the original medium was discarded, and the cells were gently washed 2–3 times with sterile PBS. The medium was then replaced with exosome-depleted medium, and the cells were cultured for an additional 24–48 h. The collected culture supernatant was subjected to sequential centrifugation at 4 °C: 300 × g for 10 min, 2,000 × g for 20 min, and 10,000 × g for 30–40 min. The final supernatant was transferred to an ultracentrifuge tube and centrifuged at 100,000 × g for 90 min at 4 °C. The supernatant was carefully discarded, and the pellet containing crude exosomes was resuspended in a large volume of pre-cooled PBS. This suspension was washed again by ultracentrifugation under the same conditions (100,000 × g, 4 °C, 90 min). Finally, the exosome pellet was resuspended in a small volume of sterile PBS or a specified buffer to obtain the purified exosome sample.

### 2.10 Flow cytometry

For cells derived from LAA tissue, prepare a cell suspension as described in Section 2.8. Transfer 5 × 10^5^–1 × 10^6^ cells to flow cytometry tubes. Add 1–2 mL staining buffer to each tube. Centrifuge at 300–400 × g for 5–8 min, then discard the supernatant. Add Fc receptor blocker and incubate at 4 °C for 10–20 min. Directly add pre-diluted CD86 antibody and CD206 antibody to the same tubes, gently vortex to mix, and incubate at 4 °C in the dark for 30 min. After incubation, add 1–2 mL staining buffer to each tube and gently pipette to mix. Centrifuge at 300–400 × g for 5–8 min, discard supernatant. Resuspend cells in 200–400 μL staining buffer and proceed immediately to flow cytometry analysis. For peripheral blood-derived cells, isolate PBMCs using Ficoll density gradient centrifugation. Add 5–10 mL PBS, centrifuge at 300 *g* for 10 min. Discard supernatant, resuspend cells in PBS, and proceed with subsequent steps as described above.

### 2.11 Primary fibroblast culture and cell experiment grouping

After anesthetizing SD rats, the hearts were rapidly excised and placed in a Petri dish. The hearts were rinsed with pre-cooled HBSS until no visible blood remained. Using scissors, the heart tissue was finely chopped, and 10 mL of trypsin was added to the tissue fragments. These were then transferred to 50 mL centrifuge tubes, sealed, and placed in a shaker at 4 °C for digestion over a period of 4 h. Following digestion, the trypsin was discarded, and the tissue was immersed in 15 mL of medium. Subsequently, 5 mL of collagen II was added to the centrifuge tubes, which were tilted, shaken, and incubated for 30 min. The mixture was pipetted, filtered through a 70 µm filter, and centrifuged at 12,000 rpm for 7 min. The supernatant was discarded, and the cell pellet was resuspended in a low-sugar, high-serum complete medium to obtain fibroblasts.

Group 1: To elucidate the function of PDGF-DD, two experimental groups were established:

Normal Group: No intervention was applied, serving as the negative control.

PDGF-DD Group: Primary atrial fibroblasts were treated with PDGF-DD at concentrations ranging from 50 ng/mL.

Group 2: In the second phase of the study, six experimental groups were designed to investigate downstream signaling pathways:

Normal Group: No intervention was applied, serving as the negative control.

Normal Group + ANG-II: Primary atrial fibroblasts were treated with 8 µM ANG-II to mimic myocardial fibrosis associated with AF.

Normal Group + ANG-II + Macrophages: Primary atrial fibroblasts were co-cultured with macrophages for 24h, and the culture system was supplemented with 8 µM ANG-II to simulate myocardial fibrosis during AF.

Normal Group + ANG-II + Macrophages + miRNA101a-shRNA: Primary atrial fibroblasts were co-cultured with macrophages for 24h, with 8 µM ANG-II added to mimic myocardial fibrosis during AF. Simultaneously, cells were transfected with miRNA101a-shRNA-specific adenovirus to achieve miRNA101a knockdown. The optimal multiplicity of infection (MOI) for transfection was determined to be 100.

Normal Group + Macrophage Group: Primary atrial fibroblasts were co-cultured with macrophages for 24 h.

Normal Group + miRNA101a-shRNA: Normal fibroblasts were transfected with miRNA101a-shRNA adenovirus.

For the LY294002 inhibitor application section, rat primary cardiac fibroblasts were used as the cell line, with the specific isolation method described above. Based on a comprehensive evaluation of multiple prior studies, a concentration of 20 μmol/L was selected for LY294002.

Each group’s design is intended to dissect the molecular mechanisms at play and validate the involvement of specific signaling pathways in myocardial fibrosis and AF.

### 2.12 Luciferase reporter assay

PDGFD 3′UTR fragments containing the predicted wild-type (Wt) or mutant (Mut) miR101a-5p binding sites were amplified and cloned into the pmirGLO luciferase vector (Promega, Madison, WI, USA). The recombinant plasmids were designated as pmirGLO-PDGFD 3′UTR-Wt/Mut, whose sequences were as follows: pmirGLO PDGFD 3′UTR-Wt: UUG​AAA​CAU​CGU​AAC​UGG​AA 3′and pmirGLO PDGFD 3′UTR-Mut: UUG​AAA​CAU​CGU​AAG​UCC​AA. About 1 × 10^4^ CFs cells were plated into 96-well plates and co-transfected with 50-nM NC-mimics or miR101a-5p and 50-nM pmirGLO-PDGFD 3′UTR-Wt/Mut using Lipofectamine 2000. The luciferase activity was measured after 48 h using the Dual-Luciferase Reporter Assay system (Promega). pTK-renilla was transfected as an internal control in each transfection. The experiments were repeated three times with triplicates.

### 2.13 RNA extraction and RT-PCR

micro RNA was extracted from the LAA tissues or cells or blood using miRcute miRNA Isolation Kit according to the manufacturer’s protocol (Tiangen biochemical technology). One microgram of miRNA from each sample was used to generate cDNAs using the RevertAidTM First Strand cDNA Synthesis Kit (#K1622; Thermo) with a special stem-loop primer for miRNAs. Based on the miR101a sequence, a stem-loop RT primer was designed with the following sequence: 5′-CTCAACTGGT GTCGTGGAGT CGGCAATTCA GTTGAG GCATCAGC-3’. Then,qRT-PCR was performed to quantify the miR101a expression level with SYBR Green PCR Master Mix (#K0223; Thermo), according to the manufacturer’s instructions. U6 was used as an internal control. The forward and reverse primers sequences for miR101a were as follows: 5′-ACA​CTC​CAG​CTG​GGT​CAG​TTA​TCA​CAG​TGC​T-3′ and 5′-TGG​TGT​CGT​GGA​GTC​G-3’. The forward and reverse primers sequences for U6 were as follows: 5′-CTCGCTTCG GCAGCACA-3′and 5′-AACGCTTCAC GAATTTGCGT-3’. The qRT-PCR was performed on BIO-RAD CFX96. The 2^−ΔΔCT^ relative quantification method was applied.

### 2.14 Bioinformatics prediction and RNA immunoprecipitation (RIP) assay

First, perform an intersection analysis using the TargetScan, miRDB, and miRTarBase databases. To detect the interaction between PDGFDD and miR101a, RNA immunoprecipitation was performed using a kit (Geneseed, Guangzhou, China) according to the manufacturer’s instructions. Briefly, 2 × 10^7^ cells were harvested and lysed with RIP lysis buffer, followed by incubation with magnetic beads conjugated with antibodies against IgG or PDGFDD. The co-precipitated RNAs were then analyzed by qRT-PCR or RT-PCR using specific primers.

### 2.15 Viral transfection

An adenovirus vector (synthesized by HanHeng Biotech) was used to establish a miR-101a knockout (si-miR-101a-shRNA) system in fibroblasts. Briefly, specific siRNA sequences or plasmids were transfected into HEK293T cells using Lipofectamine 3000. Fibroblasts were cultured in 24-well plates with polyvinylpyrrolidone (5 μg/mL) to enhance transfection efficiency. Surviving cells were further cultured and expanded for subsequent experiments. For specific transfection procedures, refer to the adenovirus instruction manual and prior technical support from our research group. For the animal adenovirus transfection section, refer to our methodology section. Selected sequences are as follows:Mature: rno-miR-101a-5p UCA​GUU​AUC​ACA​GUG​CUG​AUG​C. sponge:First reverse complement: GCA​TCA​GCA​CTG​TGA​TAA​CTG​A, then concatenate:acaggatccGCATCAGCACTGTGATAACTGAtatacGCATCAGCACTGTGATAACTGAacatcGCATCAGCACTGTGATAACTGAtcttcaGCATCAGCACTGTGATAACTGAttttttgaattcaca.

### 2.16 Cell scratch assay

First, the back of a 6-well plate was marked with straight, parallel lines using an alcohol-resistant marker, spaced approximately 0.5–1 cm apart, ensuring at least five lines crossed each well. Cardiac fibroblasts were seeded into the plates. The seeding density was determined based on cell size and growth rate, with the principle that the cells should reach 90%–100% confluence after overnight culture. After 24 h of adherence, a linear scratch was made in the cell monolayer using a sterile 100 µL pipette tip held perpendicular to the plate. Care was taken to create a straight, continuous scratch.

The cells were then gently washed three times with PBS to remove debris, and the medium was replaced with fresh serum-free medium. The cells were incubated at 37 °C in a 5% CO_2_ atmosphere. Cell migration into the scratch area was observed and photographed at 0, 24, and 48 h.

### 2.17 Statistical analysis

The experimental data were analyzed using SPSS and GraphPad Prism 10.0. A significance level of p < 0.05 was set to determine statistical significance. Each set of experimental data was independently replicated three times. All continuous variables were assessed for normality. For data following a normal distribution, results were described using the mean ± standard deviation. Conversely, data not fitting a normal distribution were summarized using the median and interquartile range. The normality of the data was evaluated using the Kolmogorov-Smirnov test, while chi-square tests were applied for categorical variables. For comparisons between two groups of normally distributed continuous variables, statistical differences were determined using t-tests and one-way ANOVA. In cases where data did not adhere to a normal distribution, the Mann-Whitney U test was employed for between-group comparisons, and the Wilcoxon signed-rank test was used for pairwise comparisons.

## 3 Result

### 3.1 miRNA101a involve of in the process of AF myocardial fibrosis

**FIGURE 1:**
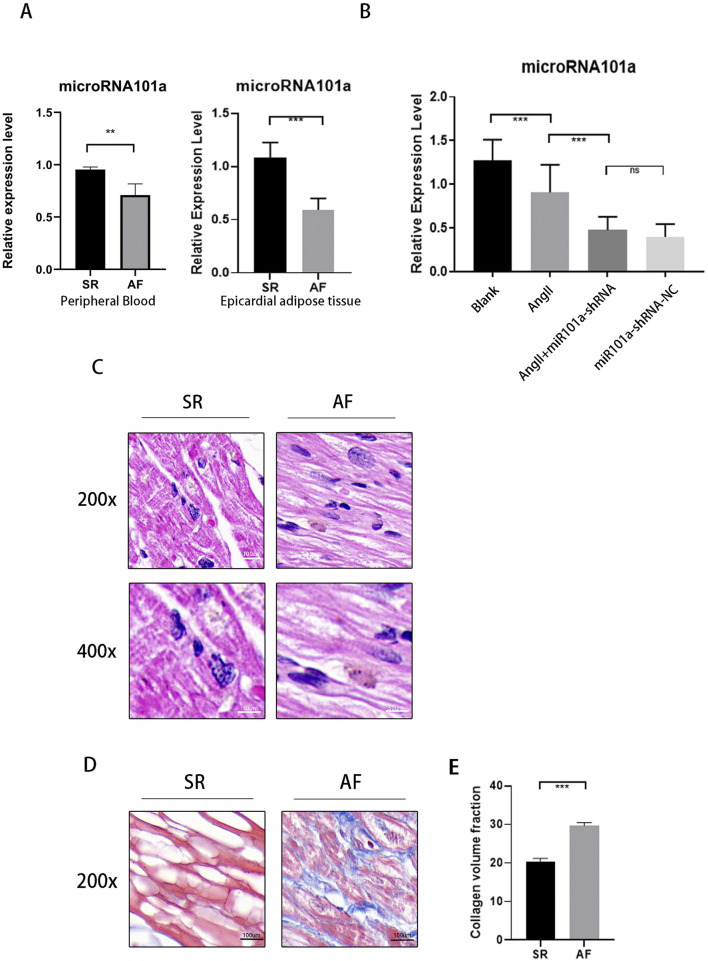
Involvement of miRNA101a in the process of AF myocardial fibrosis. **(A)** miRNA101a qPCR results of human peripheral blood (left) and Epicardial adipose tissue (right) LAA (n = 15); **(B)** miRNA101a qPCR results of rat LAA (each group n = 10); **(C)** HE staining of human tissue LAA; **(D)** Masson staining of human tissue LAA; **(E)** Masson staining analysis of fibrotic area; *indicates that the difference is statistically significant at P *<* 0.05. Each set of experimental data was independently replicated three times.

In this study, we observed (Figure 1A) a significant reduction in the expression of miRNA101a in the LAA epicardial adipose tissue and peripheral blood of patients with AF compared to the control group. This was determined by RT-PCR analysis of clinical tissue samples from the LAA, showing that miRNA101a levels were lower in the AF group than in the SR group, with the difference being statistically significant. These results are consistent with our experimental expectations. Similarly, we found the same results in animal experiments (Figure 1B): miRNA101a levels were significantly reduced in the LAA epicardial adipose tissues of rats subjected to esophageal pacing to create an AF model, compared to the control group. Additionally, we collected LAA tissues from different patient groups for HE and Masson staining. The HE staining results (Figure 1C) revealed pronounced inflammatory cell infiltration and disorganized cellular structures in the AF group compared to the SR group. Masson staining results (Figure 1D) indicated increased collagen deposition and significantly enhanced fibrosis in the myocardial tissues of the AF group. These findings suggest that myocardial fibrosis is the primary pathological mechanism underlying AF.

### 3.2 miRNA101a released by macrophage exosomes in epicardial adipose tissue targets PDGF-DD to regulate myocardial fibrosis

**FIGURE 2:**
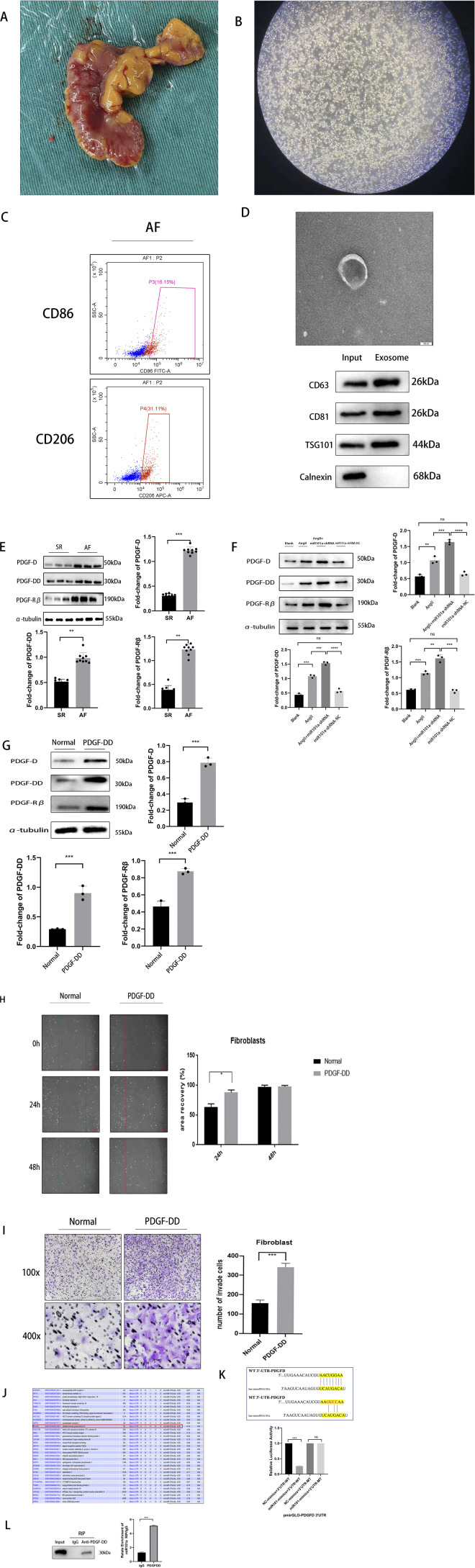
miRNA101a is released by exosomes from macrophages in epicardial adipose tissue. **(A)** Human LAA and its peripheral adipose tissue; **(B)** macrophages extracted from the peripheral adipose tissue of the LAA; **(C)** flow cytometric staining labeled with CD86 and CD206; **(D)** morphology of exosomes under electron microscope and WB detection of exosomes protein biomarkers. **(E)** Differences in PDGF family protein expression by WB assay in clinical LAA; **(F)** Differences in PDGF family protein expression in rat LAA; **(G)** Differences in PDGF family protein expression after Ang-II treatment in primary fibroblasts; **(H)** cell scratch assay; **(I)** Transwell assay to verify the migratory properties of PDGF-DD on fibroblasts; **(J)** TargetScan big data analysis; * denotes P *<* 0.05, the difference is statistically significant; **(K)** Target of miR101a in 3′UTR of PDGFD. The sequence of wild-type (WT) and mutant (MT) 3′ UTR of PDGFD gene used in luciferase assay was shown; Luciferase reporter assay showing the luciferase activities of pmirGLO vectors carrying PDGFD 3′UTR-Wt or PDGFD 3′UTR-Mut in miR101a-5p CFs cells.; **(L)** RNA Immunoprecipitation,RIP of miR101a and PDGFDD.***denotes *P* < 0.001.

To elucidate the source of miRNA101a, we successfully isolated macrophages (Figure 2B) from the adipose tissue of the LAA (Figure 2A). These macrophages were identified using flow cytometric sorting techniques by labeling with macrophage phenotypic markers, CD86 and CD206 (Figure 2C), confirming the successful extraction of EATMs. Subsequently, we isolated exosomes from the EATMs through differential centrifugation and analyzed their morphology using electron microscopy. Our observations revealed no significant differences in the morphology or particle size of the exosomes extracted from the AF group compared to the SR group,we employed WB technology to detect relevant exosomal protein markers, and experimental results confirmed our successful extraction of exosomes from epicardial adipose tissue. (Figure 2D).

Previous studies have demonstrated that the PDGF is among the key growth factors that regulate cell growth and division, influencing processes such as cell proliferation, differentiation, apoptosis, and migration (Zhao et al., 2013; Heldin and Westermark, 1989). Notably, PDGF-DD has been implicated in the progression of myocardial fibrosis. This led us to hypothesize that PDGF-DD could be a specific target of miRNA101a. To test this hypothesis, we first assessed the protein expression levels of PDGF-DD in the LAA tissues from clinical samples using WB(Western blot) analysis (Figure 2E). The results indicated that the expression levels of PDGF-D, PDGF-DD, and PDGF-Rβ were significantly higher in the LAA tissues of the AF group compared to the SR group. In our animal model, we induced AF in rats using esophageal pacing technology, successfully establishing an AF model. WB tests performed on the LAA tissues of these SD rats showed results consistent with those of the clinical samples (Figure 2F), indicating that the expression levels of PDGF-D, PDGF-DD, and PDGF-Rβ were also significantly increased in the AF group compared to the SR group. At the cellular level, we observed that the addition of PDGF-DD to cultured cardiac fibroblasts resulted in significantly elevated expression levels of PDGF-D, PDGF-DD, and PDGF-Rβ (Figure 2G).

To explore the regulatory role of PDGF-DD in cell migration, we conducted scratch assays on primary cardiac fibroblasts. The results (Figure 2H) demonstrated that PDGF-DD significantly accelerated fibroblast migration compared to the control group, with the most pronounced effect observed at 24 h. These findings suggest that PDGF-DD markedly enhances cardiac fibroblast migration, potentially exacerbating myocardial fibrosis.

Furthermore, we employed transwell assays to corroborate these observations. The results (Figure 2I) revealed that PDGF-DD intervention significantly accelerated myocardial fibroblast migration compared to the control group, as evidenced by their increased ability to penetrate the Transwell membrane. This outcome aligns with the findings from the scratch assays, reinforcing the role of PDGF-DD in promoting fibroblast migration and contributing to the progression of myocardial fibrosis.

To further investigate the interaction between miRNA101a and PDGF-DD, we utilized the miRNA target prediction database TargetScan to identify potential binding sites. Our analysis revealed (Figure 2J) that PDGF-DD is a relevant target of miRNA101a. We also utilized other databases for retrieval. Although no consistent results were found, we discovered that miR-101a may also interact with other members of the PDGF family. To verify their binding capacity, dual-luciferase reporter assays were performed (Figure 2K). The pmirGLO luciferase reporters carrying PDGFD 3′UTR-Wt/Mut were introduced together with NC/miR101a-5p into CFs cells. miR101a-5p knockdown obviously augmented the luciferase activity of vectors carrying PDGFD 3′UTR-Wt, but did not influence that of vectors carrying PDGFD 3′UTR-Mut (Figure 2K). The above findings imply that miR101a-5p binds to PDGFD 3′UTR in CFs cells. Simultaneously, we employed RIP technology for validation. Experimental results demonstrated that miR101a interacts with PDGF-DD (Figure 2L).

### 3.3 miRNA101a negatively regulates PDGF-DD expression

**FIGURE 3:**
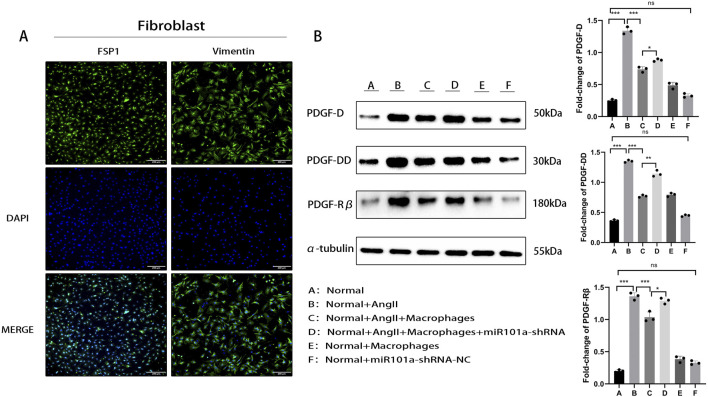
miRNA101a negatively regulates PDGF-DD expression. **(A)** Immunofluorescence staining for identification:Green:FSP, Blue:Dapi; **(B)** Cellular assay WB to verify PDGF-DD regulation; * denotes P < 0.05,the difference is statistically significant. Each set of experimental data was independently replicated three times.

We successfully isolated primary cardiac fibroblasts from the hearts of SD rats and characterized their phenotype using immunofluorescence. The experimental results showed that the nuclei of DAPI-stained cells appeared blue, while the cytoplasm was green under specific excitation wavelengths. The fibroblast phenotypic markers, Vimentin and FSP-1, were highly expressed (Figure 3A), confirming the successful extraction of high-purity cardiac fibroblasts. To investigate the regulatory role of miRNA101a on PDGF-DD, we constructed an miR101a specific knockdown adenovirus to inhibit miR101a expression. Using WB analysis, we assessed the expression of PDGF in primary atrial fibroblasts under various treatments. The results (Figure 3B) indicated that the expression levels of PDGF-DD, PDGF-D, and PDGF-Rβ were significantly elevated in the Ang-II-only intervention group compared to the control group, suggesting activation of PDGF-DD by Ang-II. Upon co-culturing with macrophages, the expression of PDGF-DD was markedly reduced. This aligns with our previous findings that miRNA101a is released by macrophage exosomes in epicardial adipose tissue, indicating that miRNA101a can inhibit PDGF-DD expression. Following transfection with the miR101a-shRNA in the co-cultured cells, we observed a slight increase in PDGF-DD expression, underscoring miRNA101a’s crucial role in negatively regulating PDGF-DD expression.

### 3.4 miRNA101a inhibits PDGF-DD-regulated fibrosis in cardiac fibroblasts

**FIGURE 4:**
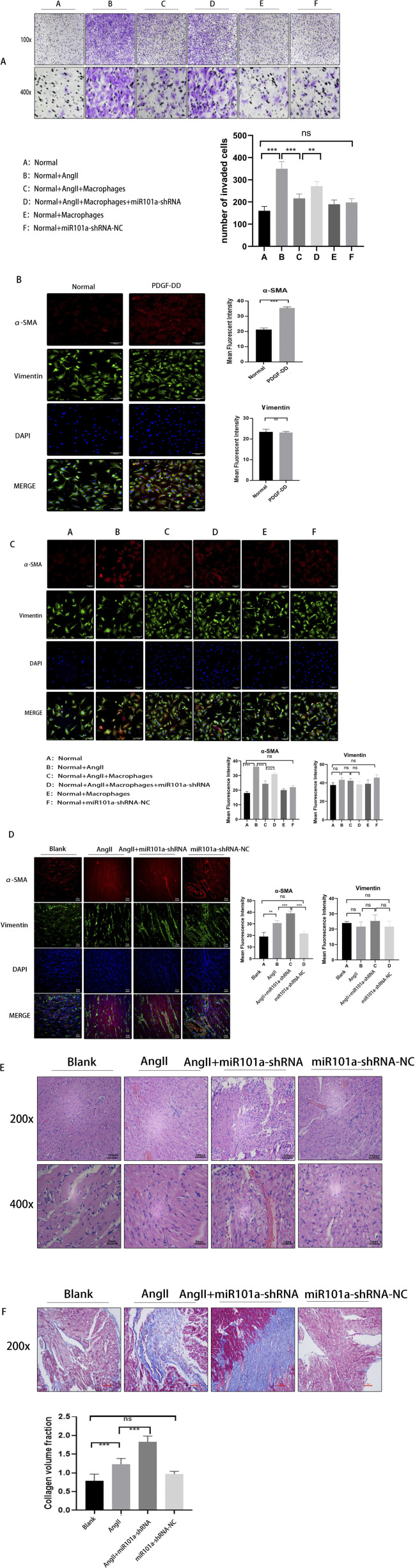
miRNA101a regulates cardiac fibroblast migration. **(A)** Cell assay Transwell; **(B)** Immunofluorescence detection of α-SMA expression after PDGF-DD stimulation:Green:Vimentin,Red:α-SMA,Blue:Dapi; **(C)** cellular assay immunofluorescence α-SMA staining (Green: Vimentin, Red: α-SMA, Blue: Dapi); **(D)** immunofluorescence α-SMA staining of rat LAA (Green: Vimentin, Red α-SMA, Blue. Dapi); **(E)** HE staining of rat LAA; **(F)** Masson staining of rat LAA; * indicates P *<* 0.05, the difference is statistically significant. Each set of experimental data was independently replicated three times.

Based on our aforementioned research findings, we confirmed the interaction between PDGF-DD and miRNA101a. To further investigate the impact of miRNA101a on the migratory and proliferative abilities of cardiac fibroblasts, we conducted Transwell assays. The results (Figure 4A) demonstrated that, compared to the control group, atrial fibroblasts treated with Ang-II exhibited enhanced migratory capacity, penetrating more readily through the chambers. However, when co-cultured with macrophages, their migratory ability was significantly reduced. In the group transfected with miR101a-shRNA, we observed a partial restoration of migratory capacity. These findings suggest that miRNA101a effectively inhibits the migration of cardiac fibroblasts regulated by PDGF-DD.

To assess the impact of PDGF-DD on cardiac fibroblast fibrosis, we employed immunofluorescence staining to detect the myofibroblast phenotypic marker α-SMA. The experimental results (Figure 4B) showed that, compared to the control group, α-SMA expression was significantly elevated following PDGF-DD stimulation, meanwhile, no significant changes were observed in vimentin, a marker of mesenchymal cells. This indicates that PDGF-DD accelerates the process of fibroblast fibrosis.

To further elucidate the specific role of miR101a, we utilized immunofluorescence staining to examine the fibroblast phenotypic marker α-SMA. The experimental results (Figure 4C) revealed that, compared to the control group, α-SMA expression was significantly increased following Ang-II stimulation, however, there was no significant change in vimentin expression. indicating an exacerbation of cellular fibrosis. However, upon co-culturing with macrophages, α-SMA expression was notably reduced. In the group subjected to miR101a-shRNA intervention, α-SMA levels increased once again. These findings suggest that miR101a plays a regulatory role in myocardial fibrosis induced by PDGF-DD in cellular experiments, effectively inhibiting the fibrosis process.

In animal experiments, we specifically targeted miR101a expression in the LAA of rats using miR101a-shRNA adenovirus. Immunofluorescence analysis (Figure 4D) revealed increased α-SMA expression and heightened fibrosis in the Ang-II intervention group compared to the control group,the expression of vimentin is also consistent with the results of the cellular experiments. Following miR101a-shRNA intervention, miR101a expression in the LAA was suppressed, leading to further elevation in α-SMA levels and suggesting increased fibrosis. These findings are consistent with our *in vitro* cellular experiments.

Additionally, we performed HE and Masson staining on the left auricle tissues of SD rats. The HE staining results (Figure 4E) indicated significant inflammatory cell infiltration and a more disorganized cardiomyocyte arrangement in the left auricle tissues of rats with AF after subcutaneous Ang-II injection and esophageal pacing, compared to the control group. This inflammatory response was further exacerbated by miR101a-shRNA intervention. Masson staining results (Figure 4F) showed that, relative to the control group, the LAA tissue of rats with AF exhibited significantly increased collagen deposition and myocardial fibrosis after Ang-II injection and esophageal pacing; this fibrosis was further aggravated by miR101a-shRNA intervention. These findings suggest that miRNA101a effectively inhibits PDGF-DD-induced myocardial fibrosis.

### 3.5 miRNA101a promotes collagen and extracellular matrix degradation under AF

**FIGURE 5:**
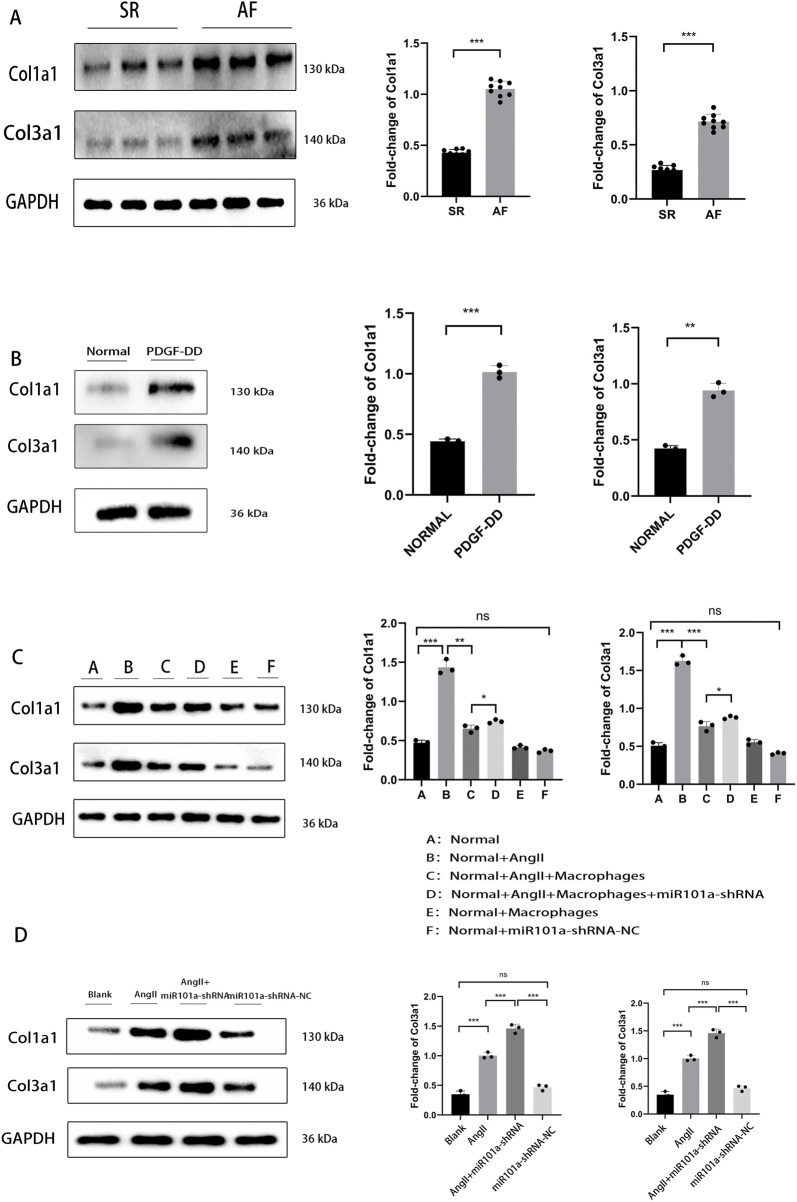
miRNA101a promotes degradation of collagen and extracellular matrix under AF. **(A)** WB detection of collagen expression in human LAA tissue; **(B)** WB detection of collagen expression after PDGF-DD stimulation; **(C)** WB detection of collagen expression in cellular experiments; **(D)** WB detection of collagen expression in rat LAA tissue; * denotes P < 0.05, the difference is statistically significant.

Atrial myocyte fibrosis is characterized by excessive collagen deposition, which led us to investigate the potential role of miR101a in regulating collagen dynamics during fibrogenesis. We initially assessed the expression of Col1a1 and Col3a1 in human LAA samples via WB. As shown in Figure 5A, both Col1a1 and Col3a1 levels were markedly elevated in the atrial fibrillation (AF) group compared to the normal SR group, indicating that AF promotes collagen accumulation and myocardial fibrosis. *In vitro*, primary fibroblasts treated with PDGF-DD exhibited significantly increased expression of Col1a1 and Col3a1 relative to controls (Figure 5B), suggesting that PDGF-DD may facilitate collagen deposition and extracellular matrix (ECM) remodeling. Moreover, Ang-II stimulation upregulated collagen expression in fibroblasts, whereas co-culture with macrophages attenuated this effect, implying active ECM degradation (Figure 5C). Notably, knockdown of miR101a using miR101a-shRNA reduced the extent of collagen degradation, pointing to a regulatory role for miR101a in this process.

In parallel *in vivo* experiments, analysis of LAA tissues from SD rats (Figure 5D) revealed a significant increase in collagen expression following Ang-II infusion compared to controls. Importantly, this effect was exacerbated upon miR101a knockdown, with further elevated levels of Col1a1 and Col3a1, consistent with enhanced collagen deposition. These findings corroborate the observations from our cellular models.

Collectively, our results demonstrate that miR101a plays a critical role in promoting collagen and ECM degradation in the setting of atrial fibrillation.

### 3.6 miRNA101a promotes macrophage phenotypic transformation under AF and plays a protective role in AF

**FIGURE 6:**
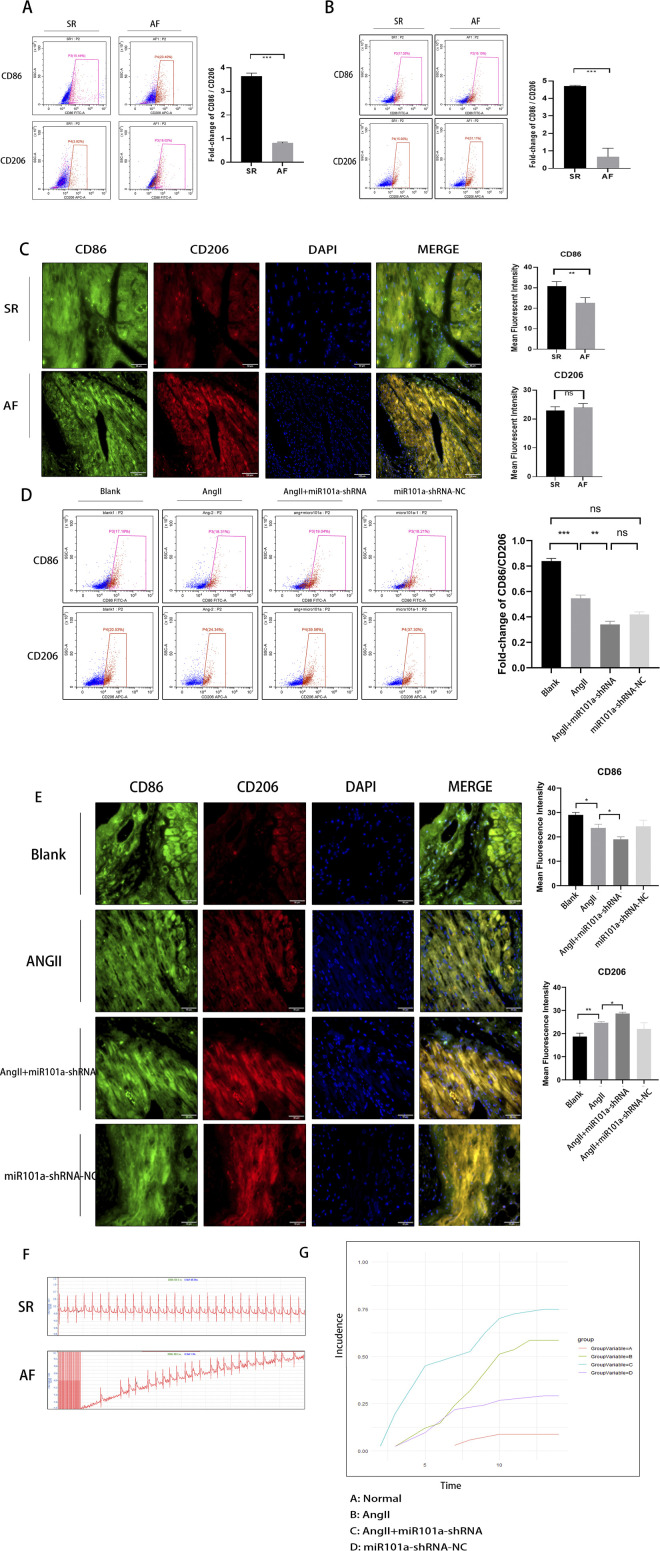
miRNA101a promotes macrophage phenotypic transformation under AF and plays a protective role. **(A)** human peripheral blood flow cytometric staining assay; **(B)** human LAA extracted macrophage flow cytometric staining; **(C)** human LAA immunofluorescence staining (Green:CD86, Red:CD206,Blue:Dapi); **(D)** rat peripheral blood flow cytometric staining assay; **(E)** rat LAA tissue immunofluorescence staining (Green:CD86,Red:CD206,Blue:Dapi); **(F)** ECG signals on rat body surface; **(G)** analysis of AF induction rate in rats within 1 week of adenoviral intervention; *Indicates P < 0.05,the difference is statistically significant.

To investigate the phenotype of epicardial adipose macrophages, we conducted a flow cytometry assay. Initially, we collected peripheral blood samples from different patient groups and assessed the phenotypic markers for M1-type macrophages (CD86) and M2-type macrophages (CD206). The results (Figure 6A) indicated that the CD86/CD206 ratio in the peripheral blood of patients with AF was significantly lower than that in the SR group. Subsequently, we extracted macrophages and performed primary cultures from the collected LAA tissues. Flow cytometry analysis revealed (Figure 6B) that the CD86/CD206 ratio in these tissues from the AF group was also significantly lower compared to the SR group. Furthermore, we conducted immunofluorescence staining on the LAA tissues. The results (Figure 6C) showed that the CD86/CD206 ratio in the left atrial tissues of patients with AF was significantly lower than in the SR group, consistent with the flow cytometry findings from peripheral blood.

These results collectively suggest a phenotypic shift in macrophages associated with AF, characterized by a lower CD86/CD206 ratio.

In our animal experiments, we established an AF model in SD rats. Blood samples were collected from these animals, and CD86 and CD206 markers were analyzed using flow cytometry. The results (Figure 6D) demonstrated that the CD86/CD206 ratio in the peripheral blood of the AF group was significantly lower compared to the control group, with a further reduction observed following miR101a-shRNA intervention. Additionally, we conducted immunofluorescence analysis on the LAA tissues of the SD rats to assess the expression levels of CD86 and CD206. The results (Figure 6E) were consistent with the flow cytometry findings from peripheral blood, confirming the phenotypic shift in macrophages associated with AF in this animal model.

We successfully developed an animal model of AF in SD rats through esophageal pacing following subcutaneous injection of Ang-II. Surface electrocardiograms (ECGs) were recorded, revealing (Figure 6F) that the ECGs of the model group displayed characteristics of AF, notably the absence of the P-wave, replaced by f-waves of varying sizes and shapes. In contrast, the control group exhibited normal SR. Further analysis of the ECG signals from the model group, collected at regular intervals, indicated (Figure 6G) that the expression of miR101a via miR101a-shRNA significantly increased the rate of AF induction and notably shortened the effective atrial refractory period in the rats. These findings suggest that miR101a may play a protective role in the development of AF.

### 3.7 EATMs-derived miR101a regulates AF through the PI3K-AKT pathway

**FIGURE 7:**
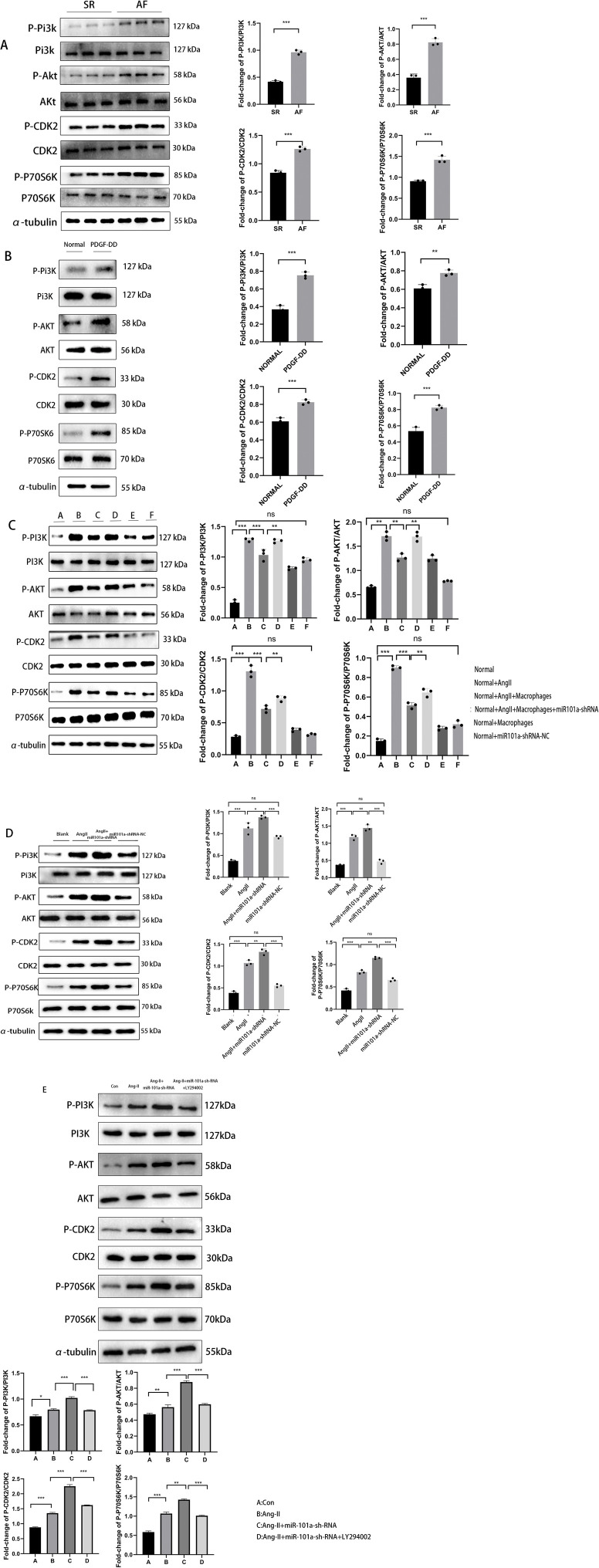
EATMs-derived miR101a regulates AF through the PI3K-AKT pathway. **(A)** WB detection of human LAA pathway protein differences; **(B)** WB detection of PDGF-DD post-stimulation pathway protein differences; **(C)** WB detection of cellular experimental pathway protein differences; **(D)** WB detection of rat LAA pathway protein differences; **(E)** WB Detection Results and Statistical Analysis: Cells Treated with the PI3K Inhibitor LY294002; * denotes P < 0.05,the difference is statistically significant. Each set of experimental data was independently replicated three times.

In our previous experiments, we found that AF occurs with an increase in collagen deposition and extracellular matrix, specifically in Col1a1,Col3a1. Previous studies (Huang et al., 2024) have demonstrated that the PI3K/AKT pathway plays a role in pulmonary fibrosis. Therefore, our next WB experiment examined the expression of PI3k/Akt pathway-related proteins under different conditions. Firstly, in clinical LAA samples, we found (Figure 7A) that the expression levels of phosphorylated PI3K, Akt, CDK2, and P70S6K proteins were significantly increased in the AF group in comparison with the SR control group, which indicated that the PI3K-AKT pathway was activated to play a role in AF.

To investigate whether PDGF-DD regulates the downstream PI3K-AKT pathway, the experimental results (Figure 7B) revealed that the expression levels of phosphorylated PI3K, Akt, CDK2, and P70S6K proteins were significantly increased by the addition of PDGF-DD stimulation compared with the control group, and this experimental result suggests that PDGF-DD directly regulates the PI3K-AKT pathway. PI3K-AKT pathway directly by PDGF-DD.

To further investigate the association between miR101a and the PI3K-Akt pathway, we employed Western blot (WB) analysis to assess the expression of pathway-related proteins in our cellular experiments. The results (Figure 7C) demonstrated that the levels of phosphorylated PI3K, Akt, CDK2, and P70S6K proteins were significantly elevated in the group stimulated with Ang-II compared to the control group. Interestingly, when macrophages were co-cultured, the expression levels of PI3K, Akt, CDK2, and P70S6K proteins were significantly reduced following Ang-II stimulation, indicating inhibition of the PI3K-Akt pathway. However, upon intervention with miR101a-shRNA, the extent of PI3K-Akt pathway inhibition in the co-culture group was diminished, and the pathway was even partially reactivated. These findings suggest that miR101a modulates the PI3K-Akt pathway, potentially affecting its activity in the presence of Ang-II and macrophage interactions.

In our *in vivo* animal experiments, we observed consistent results (Figure 7D): the expression levels of phosphorylated PI3K, Akt, CDK2, and P70S6K proteins were significantly elevated in the group treated with Ang-II compared to the control group. Furthermore, when miR101a expression was knocked down in the LAA of rats using miR101a-shRNA, the expression levels of these phosphorylated proteins increased even further, indicating enhanced activation of the PI3K-Akt pathway.

To validate the specific regulatory mechanism between miR101a and the PI3K-AKT pathway, we treated cells with the PI3K pathway-specific inhibitor LY294002. The experimental results revealed that (Figure 7E): Compared to the miR101a-shRNA transfection group, co-treatment with LY294002 significantly suppressed the expression of corresponding downstream pathways in the PI3K-AKT pathway. This further corroborates our experimental conclusion that miR101a regulates AF-induced myocardial fibrosis through the PI3K-AKT pathway.

These findings suggest that miR101a derived from EATMs plays a crucial role in regulating AF through its interaction with the PI3K-Akt pathway. Moreover, miR101a appears to influence the progression of myocardial fibrosis in AF by modulating this signaling pathway, highlighting its potential as a therapeutic target for managing Firstly, in clinical LAA samples, we found (Figure 7A) that the expression levels of phosphorylated PI3K, Akt, CDK2, and P70S6K proteins were significantly increased in the AF group in comparison with the SR control group, which indicated that the PI3K-AKT pathway was activated to play a role in AF.

## 4 Discussion

AF is the most common cardiac arrhythmia, capable of causing a variety of symptoms, including palpitations, dizziness, shortness of breath, and fatigue (Rienstra and Van Gelder, 2013). The pathogenesis of AF is complex, with myocardial fibrosis playing a crucial role in its development and maintenance. Myocardial fibrosis is primarily characterized by the abnormal proliferation of collagen and other matrix components within cardiac tissues, leading to alterations in myocardial structure and function.

Fibroblasts are central to the process of myocardial fibrosis. During AF, fibroblasts become activated and transform into myofibroblasts, which exhibit an enhanced capacity to synthesize collagen and other matrix components. These myofibroblasts secrete large quantities of ECM components, such as collagen and fibronectin (Sagris et al., 2022). The excessive accumulation of these ECM components results in changes to myocardial organization and increases the stiffness and electrical heterogeneity of the atria, thereby providing a substrate for the development and maintenance of AF. Additionally, fibroblasts contribute to the inflammatory response and fibrotic process by secreting a variety of cytokines and growth factors. This activity creates a vicious cycle that further exacerbates myocardial fibrosis.

In recent years, research into the occurrence and mechanisms of AF has intensified, yet the specific pathophysiological and molecular mechanisms remain incompletely understood. It has been demonstrated that miRNAs are involved in numerous biological processes, including development, disease progression, and evolution, and they play a pivotal role in the pathogenesis of cardiovascular diseases (Stępień E. et al., 2018). microRNA-650 can be involved in mediating the expression of TGFβ1 to maintain the contractile phenotype of aortic vascular smooth muscle cells, and play a protective role in aortic coarctation, as demonstrated by the studies of Huang et al. (2023). The study of Liao et al. (2021) confirmed that miRNA-21-5p could inhibit apoptosis of myocardial microvascular endothelial cells and promote angiogenesis after myocardial infarction by targeting silencing of cdip1. It has been shown that miRNAs expression in exosomes of patients with AF and those with SR is significantly different and may play an important biological function in atrial fibrillogenesis (Ouyang and Wei, 2021). A growing number of studies have shown that circulating miRNAs can be involved in cardiac fibrosis as well as cardiomyocyte apoptosis by regulating gene expression. Other studies have confirmed that miRNAs are closely related to structural remodeling, an important structural basis in AF (Vaze et al., 2020; Stępień E. et al., 2018; Zou et al., 2019). Previous researcher (Mun et al., 2019) showed that the expression of five miRNAs was elevated several-fold in patients with persistent atrial fibrillation compared with patients with supraventricular tachycardia. Another study found 39 miRNAs that differed between patients with AF and those with SR by examining exosomal miRNA expression (Wang S. et al., 2019). The current study has not yet studied the miRNAs in exosomes of EATMs in patients with AF in depth, so this is where our attention and interest lies.

miR101a is a small non-coding RNA widely expressed in eukaryotes. It regulates the expression of target genes by binding to their mRNA, thereby influencing processes such as cell proliferation and development (Yao et al., 2020; Yin et al., 2014; Pan et al., 2012). miRNA101a has been shown to play an important role in a variety of diseases, including tumors, cardiovascular diseases, neurological diseases, etc (Chen et al., 2019).

miRNA101a can affect processes such as proliferation, apoptosis and autophagy in cardiomyocytes by targeting and regulating the expression of certain genes (Konno et al., 2014; Xin et al., 2019). For example, it has been shown that miRNA101a can downregulate the expression of the Fos gene, thereby inhibiting the proliferation and migration of cardiomyocytes (Konno et al., 2014). In addition, miRNA101a can promote autophagy in cardiomyocytes by regulating the expression of the autophagy-related gene Beclin-1, which in turn protects cardiomyocytes from ischemia/reperfusion injury to a certain extent (Zhai et al., 2020). microRNA-101a is involved in the regulation of the expression of a wide range of cells, including such as endothelial cells, smooth muscle cells and fibroblasts (Wang et al., 2021; Lei et al., 2019; Wang N. et al., 2019; Li et al., 2021b), miRNA101a can inhibit the fibrotic process by targeting and inhibiting the expression of Col1α1 and α-SMA, reducing collagen synthesis in fibroblasts and myofibroblast differentiation (Li et al., 2021a). In addition, miRNA101a is able to affect the migration ability of fibroblasts by regulating the expression of MMPs (matrix metalloproteinases) family genes, which in turn plays an important role in tissue repair and scar formation (Beauchemin et al., 2015), given its role in regulating atrial fibrosis (a hallmark lesion of AF), we hypothesize that this molecule may also be elevated in other conditions associated with significant cardiac fibrosis, such as heart failure. We will focus on research in this area in subsequent studies.

Overall, the findings of this study indicate that miR101a may play a role in the development of AF. Thus, we hypothesize that miR101a could serve as a potential prognostic target in AF. However, further investigation is needed to explore the biological role of miR101a in exosomes to substantiate this hypothesis.

Exosomes are extracellular vesicles, ranging in diameter from 30 to 150 nm, that play a crucial role in intercellular communication. In recent years, they have garnered increasing attention in cardiovascular research due to their involvement in mediating cellular signaling, thereby influencing the progression of cardiovascular diseases (Xiang et al., 2022). A study by Olga Shaihov-Teper et al. (Shaihov-Teper et al., 2021) has demonstrated that exosomes in the epicardial adipose tissue of patients with atrial fibrillation are able to secrete more pro-inflammatory substances. By analyzing serum-derived exosomes, Yun Xie’s research team found (Xie et al., 2023) that serum exosomal miRNA expression profiles of AF patients were different from those of controls. In previous studies, we have demonstrated that miRNA101a secreted by exosomes from macrophages in epicardial adipose tissue is significantly differently expressed in patients with atrial fibrillation versus those with sinus rate, but whether it can act directly on myocardial fibroblasts needs to be further verified.

Macrophages originate from monocytes in the peripheral blood that have migrated into tissues through the blood vessels. When stimulated by injury or an inflammatory response, these circulating monocytes differentiate into macrophages. Macrophages are primarily classified into two types: classically activated macrophages (M1), which exhibit a pro-inflammatory phenotype, and alternatively activated macrophages (M2), which have an anti-inflammatory phenotype. The extent of macrophage polarization plays a critical role in the transformation of inflammatory cells and the modulation of inflammatory phenotypes (Ahangari et al., 2023), and cardiac macrophages (cMPs) are increasingly recognized as important regulators of myocardial homeostasis and disease. Studies have shown that inflammatory responses play a key role in the development and maintenance of AF, and previous studies have demonstrated that changes in macrophage phenotype and function can lead to maladaptive repair, leading to the development of chronic inflammation and pathologic fibrosis (Wu et al., 2023). Ramanujam et al. (2021) revealed that cMPs play a key role in pressure overload-induced cardiac fibrosis and dysfunctionand revealed that macrophage miR-21 is a key molecule in the pro-fibrotic role of cMPs. In other study (Wu et al., 2023) showed that macrophage M2 polarization promotes cardiac fibrosis and AF inducibility. Watson et al. (2020) in their study found that in patients with AF, atrial tissue M2 macrophage CD163+, collagen volume fraction, and gene expression of pro-collagen1 and three were elevated. In the present study, we hypothesized that miR101a might also play a role in regulating macrophage polarization. Researchers have explicitly identified the CD86/CD206 ratio as an evaluative indicator, demonstrating that it promotes macrophage polarization toward the M1 phenotype by activating the Notch signaling pathway (Hu et al., 2023). Another study also noted that the macrophage response to lipid stimulation manifests as a significant increase in the CD86/CD206 ratio (Pedersen et al., 2023). This suggests that acute exercise may exert protective effects by regulating macrophage polarization toward the M1 phenotype. In this study, we propose that the protective effect of miRNA101a is partially achieved by modulating macrophage phenotypes, specifically by suppressing the M2 phenotype. In AF patients, the M2 phenotype is inhibited, meaning the anti-inflammatory phenotype is suppressed, leading to reduced expression of the protective factor miR101a secreted by exosomes. Our findings demonstrated that the deletion of miR101a induced a shift in macrophage polarization from the M1 to the M2 phenotype. This shift was evidenced by a decreased CD86/CD206 ratio, which was confirmed through flow cytometric analysis.

PDGF, Platelet Derived Growth Factor is a biologically active cytokine produced by cells such as platelets and macrophages, which has the ability to promote cell proliferation, migration, and repair of damaged tissues. PDGF can act through the formation of dimers or more complex forms. PDGF-DD regulates myocardial fibrosis by initiating a series of downstream signaling pathways through binding to its specific receptors, PDGFR-α and PDGFR-β.

Firstly, PDGF-DD induces the proliferation and migration of fibroblasts by activating PDGFR-α/β receptors. Upon stimulation by PDGF-DD, fibroblasts exhibit accelerated proliferation and enhanced migration, contributing to the formation and progression of myocardial fibrosis. Secondly, PDGF-DD facilitates the conversion of fibroblasts into myofibroblasts, which are key players in myocardial fibrosis due to their ability to synthesize and secrete large amounts of collagen and fibronectin. Under the influence of PDGF-DD, fibroblasts transform into myofibroblasts, characterized by high expression of the myofibroblast marker α-SMA. Additionally, PDGF-DD regulates cell proliferation, differentiation, and survival by activating multiple signaling pathways, such as PI3K/Akt and ERK/MAPK. These pathways not only influence fibroblasts and myofibroblasts but also may affect the function of cardiomyocytes and endothelial cells, thereby exerting a broader impact on myocardial fibrosis. In conclusion, PDGF-DD plays a multifaceted role in the regulation of myocardial fibrosis through various mechanisms.

The involvement of PDGF-DD in mediating myocardial fibrosis has been demonstrated in our experiments to be able to be reversed by miRNA101a.It has been suggested that the PI3K pathway may be involved in the development of AF, which is expected to serve as a non-invasive molecular therapeutic strategy (Ezeani and Prabhu, 2021). the PI3K pathway affects cell proliferation and apoptosis by regulating a variety of downstream signaling molecules, such as Akt (Protein Kinase B), mTOR, and others. Secondly, the PI3K pathway is also involved in the regulation of inflammatory responses. Inflammatory factors such as TNF-α and IL-6 can exacerbate myocardial fibrosis by activating the PI3K/Akt pathway and promoting fibroblast proliferation and collagen synthesis. Studies have shown that the PI3K/Akt pathway can influence the electrophysiological properties of atrial cells by modulating the expression and function of ion channels, such as potassium channels (IKr, IKs) and sodium channels (INa).In addition, the PI3K pathway can be involved in the development and maintenance of AF by regulating calcium homeostasis and affecting the contractile function of cardiomyocytes (Ghigo et al., 2017).

In summary, the PI3K pathway is integral to the development of AF and myocardial fibrosis, influencing the behavior of cardiomyocytes and fibroblasts through a variety of mechanisms. Consequently, therapeutic interventions targeting the PI3K pathway could offer innovative approaches for the treatment of AF and its associated myocardial fibrosis.

In this study, we observed that during the onset of AF, there is a decrease in miR101a secreted by epicardial adipose macrophage exosomes. This reduction is accompanied by an increase in inflammatory factors and the activation of macrophages. This cascade leads to the activation of PDGF-DD upon binding to its receptor, PDGF-Rβ, subsequently triggering the overexpression of the PI3K-Akt pathway. This activation further stimulates downstream CDK2 signaling. Collectively, these signaling cascades contribute to the development of AF-induced myocardial fibrosis. Moreover, this process can be exacerbated by knocking down miR101a expression, highlighting its regulatory role in the progression of AF-associated myocardial fibrosis.

Our study position miR101a as a molecule with significant translational potential in myocardial fibrosis. As a therapeutic target, our data suggest that restoring miR101a expression could suppress pathological fibroblast activation by targeting PDGFD, a key mediator of macrophage-fibroblast crosstalk during cardiac remodeling (Hamid et al., 2022). Pro-inflammatory macrophages promote fibroblast differentiation in part through PDGF/PDGFRβ activation, and our identification of miR101a as an endogenous brake on this axis offers a novel anti-fibrotic strategy. This aligns with previous reports that miR101a overexpression reduces cardiac fibrosis and improves post-MI function (Pan et al., 2012; Zhou et al., 2018).

As a biomarker, the stability of miRNAs in biofluids makes miR101a an attractive candidate for non-invasive disease monitoring (Katayama et al., 2025). Reduced circulating miR101a levels have been associated with post-MI left ventricular dysfunction, suggesting its potential as a diagnostic indicator of fibrotic progression (Zhang et al., 2024).

In summary, our findings identify miR101a as a critical regulator of the macrophage-fibroblast axis in cardiac fibrosis, with dual potential as both a therapeutic target and a clinical biomarker.

## 5 Limitation and expectation

There are still some limitations in this study, firstly, we lacked transcriptomic screening and only screened the target genes of miRNAs using the BioSignal website, transcriptomic profiling of macrophages following miR101a modulation would likely reveal a broader network of direct and indirect targets. For instance, beyond its direct 3′UTR interactions, miR101a might influence downstream effectors involved in metabolic reprogramming (e.g., glycolysis-related enzymes) or additional inflammatory cascades that were not probed in our study. and secondly, the EATMs-Exo-miR101a focused in this study only explored animal *in vivo* models, and its effect was only verified in cellular and animal models, and it still has a long way to go before the human experiments are conducted. In addition, the investigation of the related molecular mechanisms is still not in-depth enough, and the next research direction will be focused on the deeper histological modifications to explore the more specific molecular mechanisms. Additionally, our selection of control groups could have been more refined. The absence of controls without other cardiac pathologies such as ventricular tachycardia or heart failure represents a limitation of our study. Furthermore, our failure to conduct long-term follow-up of patients with arrhythmias and establish a longitudinal cohort study constitutes another shortcoming. Future studies integrating RNA-seq with crosslinking immunoprecipitation (CLIP-seq) technologies will be essential to map the comprehensive miR101a interactome and to distinguish between its direct targets and indirect transcriptional consequences. Such an approach would not only validate our findings but could also uncover novel, therapeutically relevant targets in AF.

Future studies in human subjects are essential to translate our findings on miR101a into clinical applications for myocardial fibrosis. We propose a stepwise roadmap for human validation:

First, prospective cohort studies are needed to establish circulating miR101a as a diagnostic or prognostic biomarker. Recent evidence demonstrates that reduced miR101a expression is detectable in the circulation of patients with cardiovascular pathology—specifically, women with gestational hypertension showed significantly lower miR101a levels compared to healthy controls (Szczerba et al., 2025). Extending these observations to heart failure patients, future studies should correlate plasma miR101a levels with cardiac MRI-derived fibrosis burden, echocardiographic parameters (e.g., LVEF, diastolic function), and serum fibrotic markers such as PIIINP and PICP (Stienen et al., 2021). Importantly, We are working to establish a clinical trial biobank, which will be crucial for our future research plans. Second, translational pharmacology studies are required to evaluate the therapeutic potential of miR101a mimics. Key considerations include:developing targeted delivery systems to achieve cell-specific uptake in cardiac fibroblasts and/or infiltrating macrophages.

## Data Availability

The raw data supporting the conclusions of this article will be made available by the authors, without undue reservation.
