# Correction: miRNA101a secreted by EATMs regulates atrial fibrillation through the PDGF-mediated PI3K/AKT pathway

**DOI:** 10.3389/fphar.2026.1861200

**Published:** 2026-05-11

**Authors:** Zheng Sihao, Li Xiaoliang, Yue Honghua, Qin Xiaoli, Jiang Daisong, Zhang Zheng, Liang Weitao, Wu Zhong

**Affiliations:** 1 Department of Cardiovascular Surgery, West China Hospital, Sichuan University, Chengdu, Sichuan, China; 2 Department of Cardiothoracic Surgery, The first People’s Hospital of Neijiang, Neijiang, China

**Keywords:** atrial fibrillation, exosome, macrophage, miRNA101a, myocardial fibrosis

There was a typographical error; ‘higher’ was erroneously used instead of ‘lower’ when describing miRNA101a levels.

A correction has been made to the section **3 Result**, *3.1 miRNA101a involve of in the process of AF myocardial fibrosis*, Paragraph Number 1:

“In this study, we observed (Figure 1A) a significant reduction in the expression of miRNA101a in the LAA epicardial adipose tissue and peripheral blood of patients with AF compared to the control group. This was determined by RT-PCR analysis of clinical tissue samples from the LAA, showing that miRNA101a levels were lower in the AF group than in the SR group, with the difference being statistically significant. These results are consistent with our experimental expectations. Similarly, we found the same results in animal experiments (Figure 1B): miRNA101a levels were significantly reduced in the LAA epicardial adipose tissues of rats subjected to esophageal pacing to create an AF model, compared to the control group. Additionally, we collected LAA tissues from different patient groups for HE and Masson staining. The HE staining results (Figure 1C) revealed pronounced inflammatory cell infiltration and disorganized cellular structures in the AF group compared to the SR group. Masson staining results (Figure 1D) indicated increased collagen deposition and significantly enhanced fibrosis in the myocardial tissues of the AF group. These findings suggest that myocardial fibrosis is the primary pathological mechanism underlying AF.”

The original article has been updated.

